# The second complete mitochondrial genome of *Alphitobius diaperinus* Panzer, 1797 (Coleoptera: Tenebrionidae): investigation of intraspecific variations on mitochondrial genome

**DOI:** 10.1080/23802359.2020.1797575

**Published:** 2020-07-23

**Authors:** Ki-Jeong Hong, Woong Ki, Hyobin Lee, Jongsun Park, Wonhoon Lee

**Affiliations:** a Department of Plant Medicine, Sunchon National University, Suncheon, Republic of Korea; b Department of Plant Medicine, Gyeongsang National University, Jinju, Republic of Korea; c InfoBoss Inc., Seoul, Republic of Korea; d InfoBoss Research Center, Seoul, Republic of Korea; e Institute of Agriculture and Life Science, Gyeongsang National University, Jinju, Republic of Korea

**Keywords:** Mitochondrial genome, *Alphitobius diaperinus*, Coleoptera, intraspecific variations, South Korea

## Abstract

We have determined the second mitochondrial genome of *Alphitobius diaperinus* Panzer, 1797 collected in Gyeonggi-do, Republic of Korea. The circular mitogenome of *A. diaperinus* is 15,512 bp long which is slightly longer than that of the previous mitogenome of *A. diaperinus*. It includes 13 protein-coding genes, two ribosomal RNA genes, and 22 transfer RNAs. The base composition was AT-biased (72.4%). Intraspecific variation between two mitogenome of *A. diaperinus* was investigated: one SNP and one INDEL were identified, presenting the low level of intraspecific variations on mitochondrial genome.

In Korea, the lesser mealworm, *Alphitobius diaperinus* Panzer, 1797 (Coleoptera: Tenebrionidae), is one of the significant pests in the poultry industry (Axtell and Arends 1990). Recently, this beetle has infected in broiler chicken houses, resulting destruction of the insulate facilities of poultry houses, reduction of chick’s overall performance, and transmission of pathogenic organisms which cause substantial economic losses for poultry producers (Nguyen et al. 2019).

To investigate intraspecific variations of its mitochondrial genome, we completed the second mitogenome of *A. diaperinus*, collected in Munsan-eup, Paju-si, Gyeonggi-do, Republic of Korea (37°88′99″N, 126°77′42″E; the specimen and its DNA were deposited at the Sunchon National University, Korea, Accession number: 190925HK0010). DNA was extracted using DNeasy Blood &Tissue Kit (QIAGEN, Hilden, Germany). Raw sequences generated from Illumina HiSeqX (Macrogen, Korea) were filtered by Trimmomatic 0.33 (Bolger et al. 2014) and *de novo* assembled by Velvet 1.2.10 (Zerbino and Birney 2008), SOAPGapCloser 1.12 (Zhao et al. 2011), BWA 0.7.17 (Li 2013), and SAMtools 1.9 (Li et al. 2009) under the environment of Genome Information System (GeIS; http://geis.infoboss.co.kr/). Geneious R11 11.1.5 (Biomatters Ltd, Auckland, New Zealand) was used to annotate based on the previous mitogenome of *A. diaperinus* (MT165524; Hong et al. 2020).

*A. diaperinus* mitogenome (GenBank accession is MT610905) is 15,512 bp long, which is slightly longer than that of the previous mitogenome of *A. diaperinus.* It contains 13 protein-coding genes (PCGs), 37 tRNAs, two rRNAs and its AT ratio is 72.4%. Gene order is conserved among 24 available Tenebrionidae mitogenomes.

Based on comparison of the two mitogenomes of *A. diaperinus*, we identified one SNP and one INDEL located in *NAD4* and AT-rich regions, respectively. One SNP is non-synonymous SNP changing 142^nd^ amino acid of *NAD4* from methionine to valine. Number of intraspecific variations identified in this study is similar to that of *Laodelphax striatellus* between D5 and D7 isolates (Seo, Jung, et al. 2019) but much less than those of *Hipparchia autonoe* (Lee et al. 2020), *Aphis gossypii* (Park, Xi, Kim, et al. 2019) (Bae et al. accepted), *Laodelphax striatellus* (Park, Jung, et al. 2019; Seo, Jung, et al. 2019), *Nilaparvata lugens* (Choi et al. 2019; Park, Kwon, et al. 2019; Choi et al. 2020), *Spodoptera frugiperda* (Seo, Lee, et al. 2019), and *Chilo suppresallis* (Park, Xi, Kwon, et al. 2019).

We inferred phylogenetic relationship of 25 Tenebrionidae mitogenomes, including two *A. diaperinus* mitogenome based on alignment of complete mito genomes by MAFFT 7.450 (Katoh and Standley 2013) after rearranging mitogenome sequences as same as the previous study (Hong et al. 2020). Bootstrapped neighbor joining, maximum likelihood, and Bayesian inference phylogenetic trees were constructed by MEGA X (Kumar et al. 2018) and Mr. Bayes (Huelsenbeck and Ronquist 2001). It displays that two mitogenomes of *A. diaperinus* were clustered and three neighbor tribes were also clustered in one clade as same as the previous study (Hong et al. 2020; Figure 1).

**Figure 1.**
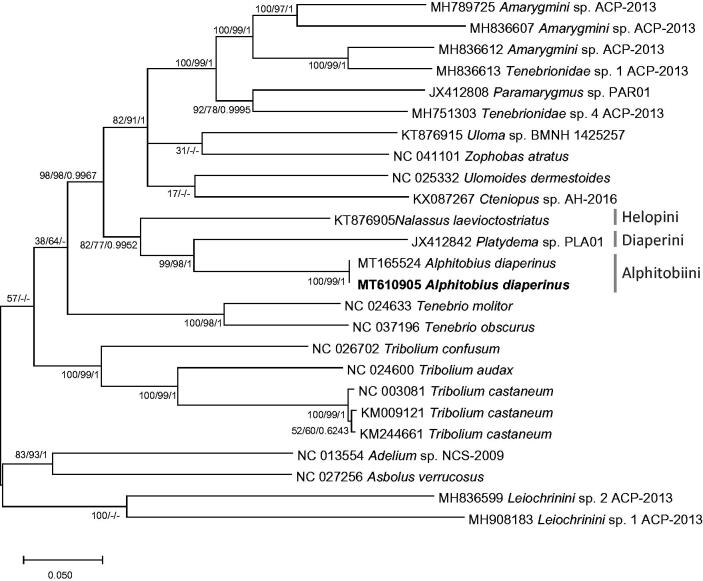
Bayesian inference (1,000,000 generations), maximum likelihood (1,000 bootstrap repeats), and neighbor joining (10,000 bootstrap repeats) phylogenetic trees of 24 Tenebrionidae mitochondrial genomes: *Alphitobius diaperinus* (MT610905 in this study and MT165524), *Amarygmini* sp. (MH789725; Partial mitochondrial genome; MH836607, and MH836612), *Tenebrionidae* sp. (MH836613 and MH751303), *Paramarygmus* sp. (JX412808; Partial mitochondrial genome), *Cteniopus*sp. (KX087267; Partial mitochondrial genome), *Zophobas atratus* (NC_041101), *Uloma* sp. (KT876915), *Ulomoides dermestoides* (NC_025332), *Nalassus laevioctostriatus* (KT876905), *Platydema* sp. (JX412842), *Tenebrio molitor* (NC_024633), *Tenebrio obscurus* (NC_037196), *Tribolium confusum* (NC_026702), *Tribolium audax* (NC_024600), *Tribolium castaneum* (NC_003081, KM009121, and KM244661), *Adelium* sp. (NC_013554), *Asbolus verrucosus* (NC_027256), and *Leiochrinini* sp. (MH836599 and MH908183). Phylogenetic tree was drawn based on maximum likelihood tree. The numbers above branches indicate bootstrap support values of maximum likelihood and neighbor joining phylogenetic trees and posterior probability value of Bayesian inference, respectively. Tribe names were displayed as light gray color and subfamily names were written as gray color.

## Data Availability

The data that support the findings of this study are openly available in NCBI (National Center for Biotechnology Information) at https://www.ncbi.nlm.nih.gov/nuccore/MT610905.

## References

[CIT0001] Axtell RC, Arends JJ. 1990. Ecology and management of arthropod pests of poultry. Annu Rev Entomol. 35:101–125. 2405769 10.1146/annurev.en.35.010190.000533

[CIT0302] Bae Y, Park J, Iee w. (accepted). The complete mitochondrial genome of Aphis gossypii Glover, 1877 (Hemiptera: Aphididae) isolated from Plantago asiatica in Korea. DOI:10.1080/23802359.2020.1792366 PMC7782666 33457986

[CIT0002] Bolger AM, Lohse M, Usadel B. 2014. Trimmomatic: a flexible trimmer for Illumina sequence data. Bioinformatics. 30(15):2114–2120. 24695404 10.1093/bioinformatics/btu170 PMC4103590

[CIT0003] Choi NJ, Lee B-C, Park J, Park J. 2019. The complete mitochondrial genome of *Nilaparvata lugens* (Stål, 1854) captured in China (Hemiptera: Delphacidae): investigation of intraspecies variations between countries. Mitochondrial DNA Part B. 4(1):1677–1678.

[CIT0004] Choi NJ, Lee B-C, Park J, Park J. 2020. The complete mitochondrial genome of *Nilaparvata lugens* (Stål, 1854) captured in Guangxi province, China (Hemiptera: Delphacidae): identification of the origin of *N. lugens* migrated to Korea. Mitochondrial DNA Part B. 5(2):1960–1961.

[CIT0005] Hong K-J, Ki W, Park D-S, Yang B-K, Lee H, Park J, Lee W. 2020. The complete mitochondrial genome of *Alphitobius diaperinus* Panzer, 1797 (Coleoptera: Tenebrionidae. ). Mitochondrial DNA Part B. 5(3):2291–2293. 33367012 10.1080/23802359.2020.1772684 PMC7510575

[CIT0006] Huelsenbeck JP, Ronquist F. 2001. MRBAYES: Bayesian inference of phylogenetic trees. Bioinformatics. 17(8):754–755. 11524383 10.1093/bioinformatics/17.8.754

[CIT0007] Katoh K, Standley DM. 2013. MAFFT multiple sequence alignment software version 7: improvements in performance and usability. Mol Biol Evol. 30(4):772–780. 23329690 10.1093/molbev/mst010 PMC3603318

[CIT0008] Kumar S, Stecher G, Li M, Knyaz C, Tamura K. 2018. MEGA X: molecular evolutionary genetics analysis across computing platforms. Mol Biol Evol. 35(6):1547–1549. 29722887 10.1093/molbev/msy096 PMC5967553

[CIT0009] Lee Y-D, Lee J, Kim D-S, Park J, Xi H, Roh J, Kim D-S, Nam SJ, Kim S-K, Song J-Y, et al. 2020. The complete mitochondrial genome of *Hipparchia autonoe* (Esper, 1783)(Lepidoptera: Nymphalidae): investigation of intraspecific variations on mitochondrial genome. Mitochondrial DNA Part B. 5(2):1542–1544.

[CIT0010] Li H. 2013. Aligning sequence reads, clone sequences and assembly contigs with BWA-MEM. arXiv. 13033997.

[CIT0011] Li H, Handsaker B, Wysoker A, Fennell T, Ruan J, Homer N, Marth G, Abecasis G, Durbin R, 1000 Genome Project Data Processing Subgroup 2009. The sequence alignment/map format and SAMtools. Bioinformatics. 25(16):2078–2079. 19505943 10.1093/bioinformatics/btp352 PMC2723002

[CIT0012] Nguyen N, Yang B-K, Lee J-S, Yoon J-U, Hong K-J. 2019. Infestation status of the darkling beetle (*Alphitobius diaperinus*) in Broiler chicken houses of Korea. Korean J Appl Entomol. 58(3):189–196.

[CIT0013] Park J, Jung JK, Ho Koh Y, Park J, Seo BY. 2019. The complete mitochondrial genome of *Laodelphax striatellus* (Fallén, 1826)(Hemiptera: Delphacidae) collected in a mid-Western part of Korean peninsula. Mitochondrial DNA Part B. 4(2):2229–2230. 33365487 10.1080/23802359.2019.1623112 PMC7687552

[CIT0014] Park J, Kwon W, Park J, Kim H-J, Lee B-C, Kim Y, Choi NJ. 2019. The complete mitochondrial genome of *Nilaparvata lugens* (stål, 1854) captured in Korea (Hemiptera: Delphacidae). Mitochondrial DNA Part B. 4(1):1674–1676.

[CIT0015] Park J, Xi H, Kim Y, Park J, Lee W. 2019. The complete mitochondrial genome of *Aphis gossypii* Glover, 1877 (Hemiptera: Aphididae) collected in Korean peninsula. Mitochondrial DNA Part B. 4(2):3007–3009. 33365831 10.1080/23802359.2019.1666051 PMC7706777

[CIT0016] Park J, Xi H, Kwon W, Park C-G, Lee W. 2019. The complete mitochondrial genome sequence of Korean *Chilo suppressalis* (Walker, 1863) (Lepidoptera: Crambidae). Mitochondrial DNA Part B. 4(1):850–851.

[CIT0017] Seo BY, Jung JK, Ho Koh Y, Park J. 2019. The complete mitochondrial genome of *Laodelphax striatellus* (Fallén, 1826)(Hemiptera: Delphacidae) collected in a southern part of Korean peninsula. Mitochondrial DNA Part B. 4(2):2242–2243. 33365493 10.1080/23802359.2019.1624645 PMC7687393

[CIT0018] Seo BY, Lee G-S, Park J, Xi H, Lee H, Lee J, Park J, Lee W. 2019. The complete mitochondrial genome of the fall armyworm, *Spodoptera frugiperda* Smith, 1797 (Lepidoptera; Noctuidae), firstly collected in Korea. Mitochondrial DNA Part B. 4(2):3918–3920. 33366251 10.1080/23802359.2019.1688119 PMC7707783

[CIT0019] Zerbino DR, Birney E. 2008. Velvet: algorithms for *de novo* short read assembly using de Bruijn graphs. Genome Res. 18(5):821–829. 18349386 10.1101/gr.074492.107 PMC2336801

[CIT0020] Zhao QY, Wang Y, Kong YM, Luo D, Li X, Hao P. 2011. Optimizing de novo transcriptome assembly from short-read RNA-Seq data: a comparative study. BMC bioinformatics. 12(14):S2. 10.1186/1471-2105-12-S14-S2 PMC3287467 22373417

